# Osteopetrosis: a rare case of portal hypertension

**DOI:** 10.2144/fsoa-2022-0028

**Published:** 2022-11-15

**Authors:** Amal Khsiba, Sahar Nasr, Lamine Hamzaoui, Moufida Mahmoudi, Asma Ben Mohamed, Manel Yaakoubi, Mouna Medhioub, Mohamed Moussadek Azouz

**Affiliations:** 1 Department of Gastroenterology & Hepatology, Mohamed Tahar Maamouri University Hospital, Faculty of Medicine of Tunis, Tunis El Manar University, Nabeul, Tunisia

**Keywords:** anemia, ascites, bone marrow transplantation, fracture, hematopoiesis, osteopetrosis, portal hypertension, splenomegaly

## Abstract

Osteopetrosis is a rare genetic bone disorder characterized by a defect in osteoclasts recruitment and function. Its manifestations are numerous and they mainly include skeletal and dental deformities, cranial nerve entrapment and infections. Over time, osteoclastic expansion invades bone marrow leaving little space for hematopoietic cells. As a result, extramedullary hematopoiesis takes place in the reticular system mainly in the spleen and liver. In these patients, portal hypertension can occur as a result of extramedullary hematopoiesis associated splenomegaly. We are reporting in this article a rare case of spontaneous bacterial peritonitis associated with portal hypertension in a patient with osteopetrosis.

## Short abstract

Osteopetrosis is a rare genetic bone disorder characterized by a defect in the recruitment of osteoclasts, a type of bone cell that breaks down bone tissue. We are reporting a rare case of intra-abdominal fluid build-up as a complication of osteopetrosis.

Osteopetrosis is a rare genetic bone disorder characterized by a defect in osteoclasts recruitment and function [1]. This results in high bone density making them fragile and easily prone to fractures. The clinical spectrum of this condition goes from mild to rapidly fatal forms. Its manifestations are numerous and they mainly include skeletal and dental deformities, cranial nerve entrapment and infections [1]. Over time, as the osteoclastic expansion invades bone marrow, space for hematopoietic cells shrinks considerably and patients start developing hematological disorders [2]. As a result, extramedullary hematopoiesis (EMH) takes place in the reticular system mainly in the spleen and liver. Once the splenic blood flow exceeds hepatic draining capacity, portal hypertension (PHT) occurs causing a myriad of clinical manifestations and life threatening complications. Although described more than a century ago, certain aspects of osteopetrosis natural history remain unknown [1]. We are reporting the first case of spontaneous bacterial peritonitis associated with PHT in a patient with osteopetrosis. The aim of this paper is to draw clinicians attention to the fact that PHT and ascites can be complications of osteopetrosis and to discuss potential explanations of this association.

## Case summary

A 36-year-old patient with osteopetrosis was admitted to our gastroenterology unit with an abdominal distension and lower limbs edema.

Her past medical history included partial loss of sight, chronic anemia, several bone fractures (shoulder, wrist, hip, foot) and femoral osteomyelitis. The patient was receiving folic acid supplementation and red blood cell transfusions in case of symptoms of anemia or when the hemoglobin level drops below 7 g/dl.

**Figure 1.**
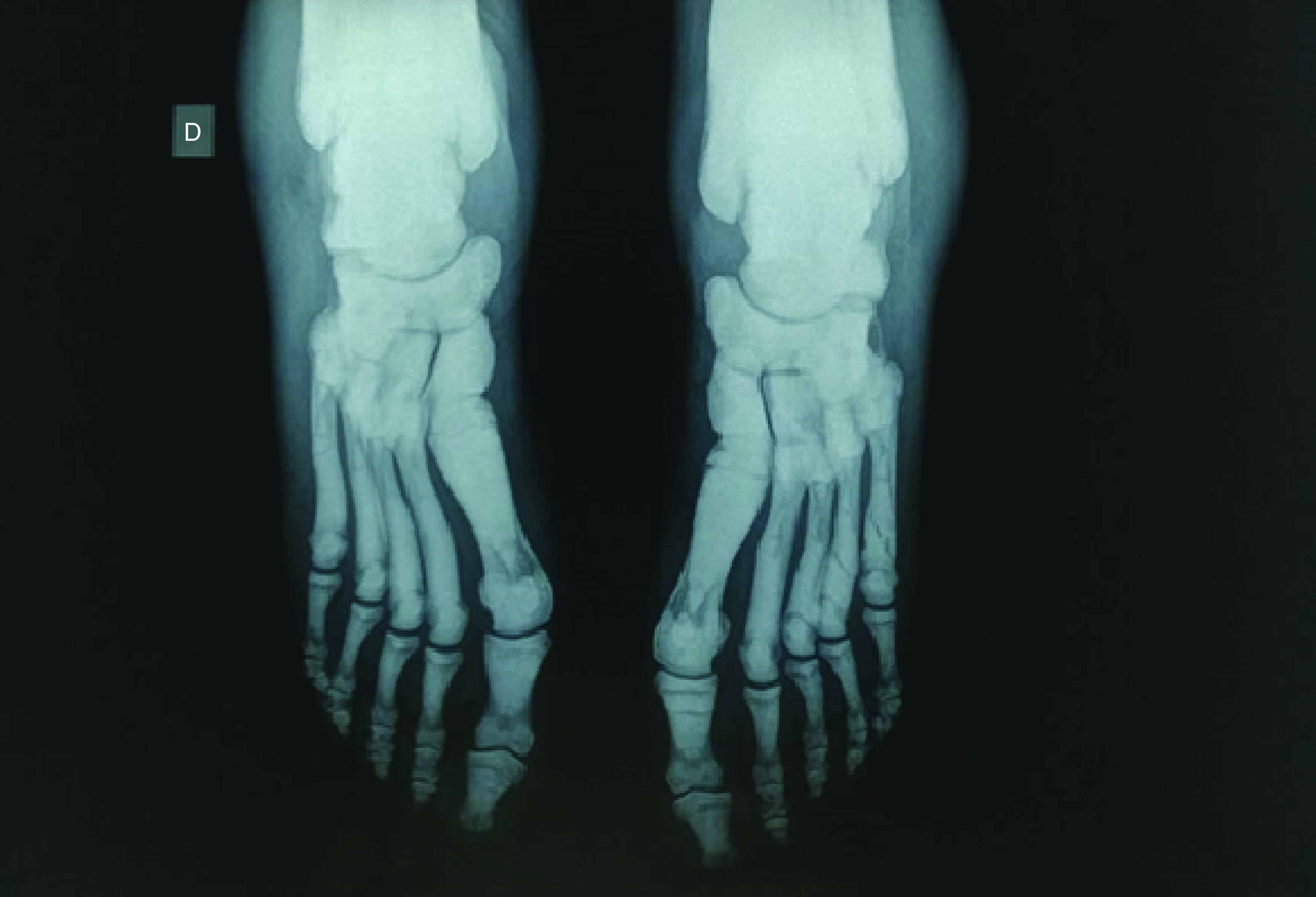
Symmetrical bilateral bone in bone aspect with multiple old phalanx and metatarsal fractures.

On admission, the patient was awake and cooperative. Her vital signs were within normal range. Her temperature was 38.3°C. The patient had a dysmorphic facial features with hypertelorism, bilateral exophthalmos and bilateral valgus knee deformities. Abdominal examination revealed massive ascites, dilated abdominal wall veins and a palpable spleen nearly 8 cm below costal margin. The liver had a smooth edge and was palpable 2 cm below costal margin. The lower extremities were edematous. The rest of the physical examination was unremarkable. Generalized ostesclerosis was detected in the chest radiography (Figure 1). The complete blood count showed hypochromic microcytic anemia, thrombocytopenia and elevated white blood cells (WBC). The blood smear showed normal WBC count and elevated count of erythroblasts.

**Figure 2.**
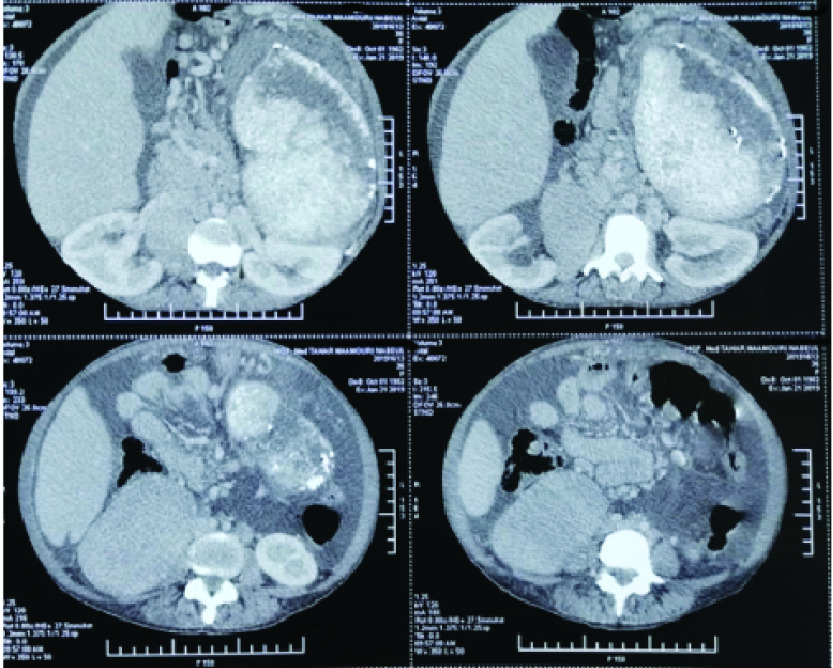
Computed tomography scan showing ascites, portosystemic collateral circulation, homogenous hepatomegaly, heterogeneous splenomegaly containing capsular and intrasplenic calcifications and segmental infarction areas. Computed tomography scan also shows a homogeneously enhanced right paravertebral mass measuring 14 × 9 × 7 cm.

The rest of the laboratory data revealed anicteric cholestasis (a fourfold increase in alkaline phosphatase), hyperferritinemia (2000 ng/ml), normal prothrombin time, hypoalbuminemia (34 g/l) with no other abnormalities at the protein electrophoresis test. Ascitic paracentesis showed a purulent fluid and the analysis revealed elevated total protein (20.5 g/l). The serum-to-ascites albumin ratio was low (7.8) and the WBC count in ascites was 750/mm^3^ with 75% of neutrophils. Both cytology and mycobacterial cultures were negative. The patient was considered to have spontaneous bacterial peritonitis. Intravenous cefotaxim was started immediately and switched to imipeneme 48 h later owing to the WBC fluid count elevation. Upper GI endoscopy did not show signs of PHT. Abdominal CT scan showed homogenous hepatomegaly with a liver span of 25 cm and a splenomegaly measuring 18 cm longitudinally and containing capsular and intrasplenic calcifications. CT imaging also revealed massive ascites with portosystemic collateral circulation and a homogeneously enhanced right paravertebral mass measuring 14 × 9 × 7 cm with several bilaterial paravertebral masses in the chest (Figure 2). These masses were considered to be secondary to peritoneal and intrathoracic EMH. No obstruction of the portal vein, hepatic veins or the inferior vena cava was found on Doppler sonography. Viral hepatitis B and C serology were negative liver related autoantibodies were negative also. Diastolic and systolic dysfunction were ruled out with transthoracic echocardiography. Overall, our patient had non-cirrhotic PHT. Although the most likely diagnosis seemed to be EMH associated PHT, we had decided to perform liver biopsy once the infection is managed and the ascites is mobilized. About 2 weeks after her admission, the patient presented a right subtrochanter fracture and was operated on. She passed away of pulmonary embolism.

## Discussion

Osteopetrosis is a hereditary disease characterized by reduced osteoclastic activity. This results in an unequal balance between bone growth and elimination. Although dense, the bones end up brittle and easily prone to fractures. Osteoclasts need to achieve maturity to become able of bone resorption. This path includes several steps, the last of which is acidification of the bone in order to dissolve the mineral matrix [2]. The genetic defect in osteopetrosis consists in loss of function mutations affecting the acidification step [2]. Osteopetrosis is characterized by a wide clinical spectrum going from mild to rapidly fatal. Its clinical manifestations include skeletal and dental abnormalities, infections and disruption of mineral homeostasis [1].

The diagnosis of osteopetrosis usually relies on the presence of typical clinical and radiological features [3]. Typical radiological findings include diffuse osteosclerosis, cortical thickness, reduction in the medullary canal diameter and a ‘bone-in-bone’ aspect in the spine and the phalanges. In the absence of these typical features, laboratory testing for an elevated creatinine kinase BB and tartrate-resistant acid phosphatase can yield supplementary arguments for the diagnosis [3,4]. Recent guidelines recommended genetic testing when osteopetrosis is highly suspected as it can provide critical data regarding prognosis, clinical associations and management decisions [1].

In severe osteopetrosis, hematological complications commonly occur owing to medullary cavities invasion by the bone expansion process leading to bone marrow failure. In the early stages, patients start developing chronic anemia. As a result, anemia induced EMH occur, as attested by the presence of early myeloid cells on peripheral blood smear in this case. EMH classically results in a myriad of complications such as hepatosplenomegaly and paravertebral and presacral mass [5]. Our patient developed a less common complication of EMH, non-cirrhotic, non-obstructive PHT. To the best of our knowledge, only three similar cases were reported in English literature. The first case was revealed by abdominal collateral circulation and PHT was confirmed with hepatic vein and abdominal collateral pressure estimation [6]. In the second case, PHT was incidentally diagnosed. The patient was 15 years old and presented mild ascites and splenomegaly at abdominal doppler ultrasonography [7]. Interestingly, the third case was revealed by esophageal varices related hematemesis. This patient responded well to octreotide and later she was started on nadolol, furosemide and spironolactone for mild ascites [8]. In fact, several case reports of EMH associated PHT have been reported in literature. In most of them, EMH was induced by idiopathic myelofibrosis where PHT is thought to develop in nearly one of every ten patients [9]. The mechanisms of PHT in idiopathic myelofibrosis are both multiple and overlapping. They include sinusoids infiltration, PV thrombosis and increased splenic venous outflow [9]. The latter is thought to be the main mechanism leading to PHT in osteopetrosis. The increased flow through the spleen would overwhelm the hepatic drainage capacity resulting in PHT. Sinusoidal invasion by hematopoietic foci might also play a role in PHT development, although we would need a liver biopsy to confirm it [10]. It is worth noting that ascites in this case might have partially resulted from peritoneal EMH that seemed very likely based on the patient’s CT scan findings. In fact, cases of peritoneal EHM revealed by ascites were previously reported in literature, although rare and exclusively associated to myeloproliferative diseases [10].

On the other hand, spontaneous bacterial peritonitis is unique to this case. It is well known that patients with osteopetrosis are prone to infections to different extent depending on disease phenotype [1]. Osteomyelitis is the most common infection, it emanates from leukopenia and compromised vascularity and local immune response. The mainly affected bones are the maxillofacial skeleton followed by scapula and long bones [1,11–13]. However, extra skeletal infections are very rare, they are mainly seen in patients with marrow failure and predominated by opportunist infections. The exact mechanism of spontaneous bacterial peritonitis in our patient is beyond this study. Yet, we believe that it might have been the result of several factors including a compromised immune system, bacteria inoculation during the repetitive transfusions or even bacterial load released from insufficiently treated old osteomyelitis sites.

A detailed discussion about the indications of hematopoietic cell transplantation and other management methods for specific situations is beyond the scope of this review. Yet, it is worth mentioning that the optimal treatment of osteopetrosis should be tailored to each patient and is best started with a thorough evaluation for potential manifestations and complications. Routine dental evaluation and maintenance of proper oral hygiene is required in these patients and can help prevent several complications [1,3]. Fractures and arthritis should be treated by an experienced orthopedic surgeon as the postoperative course might be complicated with several complications including defective remodeling, delayed union and osteomyelitis. On the other hand, hematopoietic cell transplantation is reserved for cases of malignant infantile osteopetrosis and can be discussed in cases of neurological involvement and unremitting pain [1,3].

## Conclusion

To sum up, PHT complicating EMH is rare in patients with osteopetrosis. Yet, this entity might result in severe complications such as gastro-intestinal bleeding, and in the case of our patient, massive ascites and spontaneous bacterial peritonitis. Awareness of this condition is crucial for a better understanding of osteopetrosis course and progression.

**Executive summary**

- Osteopetrosis is a rare genetic bone disorder characterized by a defect in osteoclasts recruitment and function.
- In severe osteopetrosis, hematological complications commonly occur owing to medullary cavities invasion.
- Extramedullary hematopoiesis can occur in patients with osteopetrosis as a result of bone marrow failure.
- In patients with osteopetrosis, extramedullary hematopoiesis can lead to splenomegly, an increased splenic venous outflow and thus portal hypertension.
